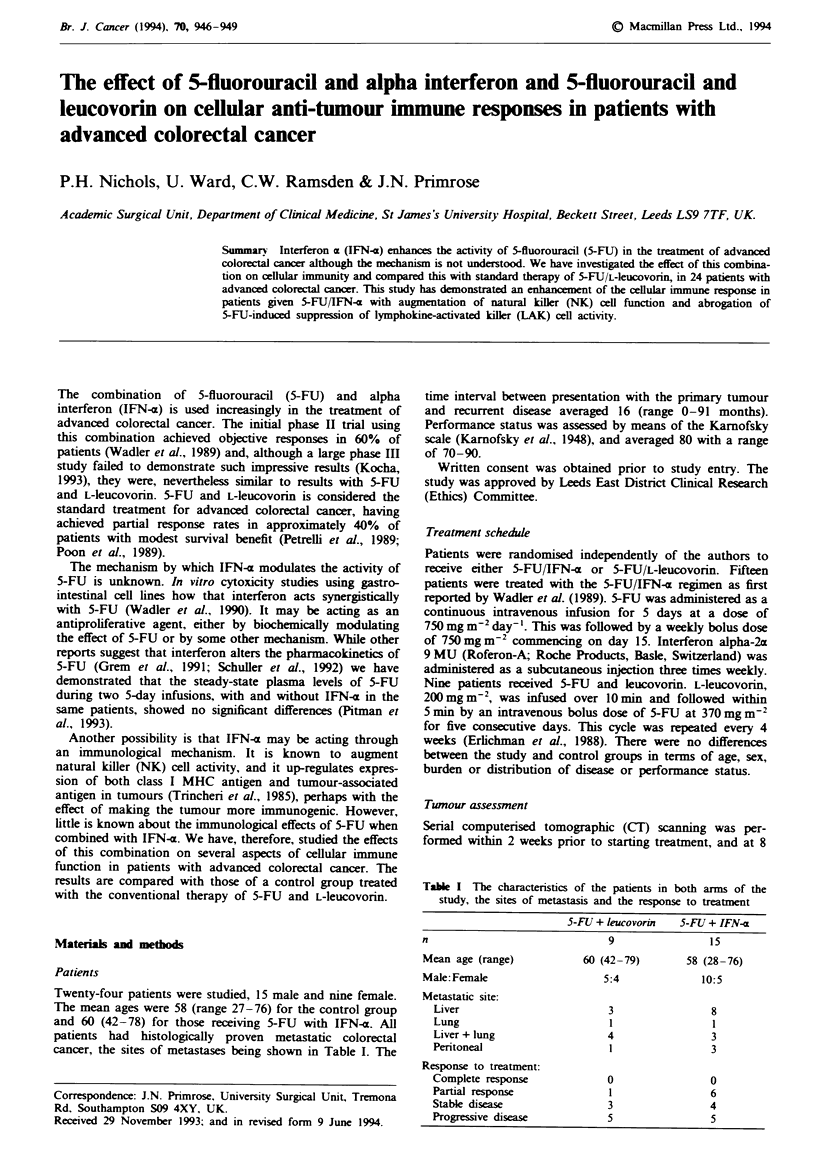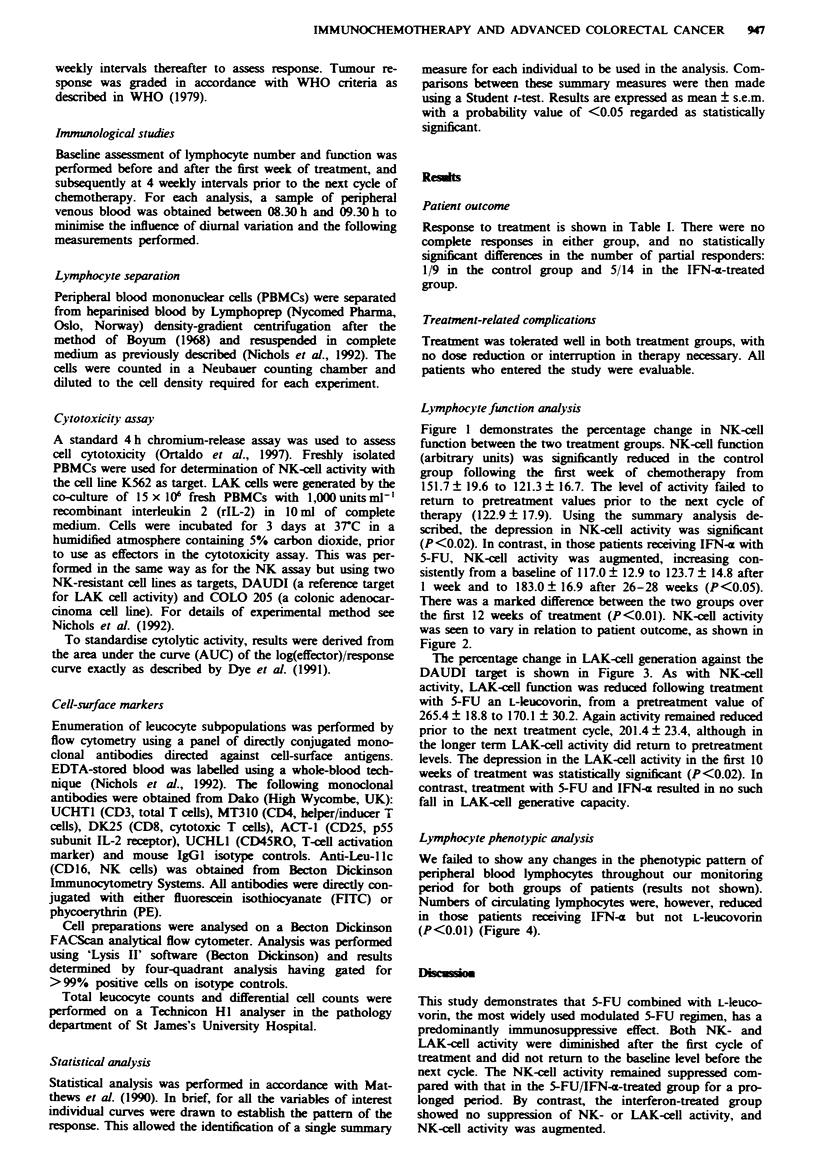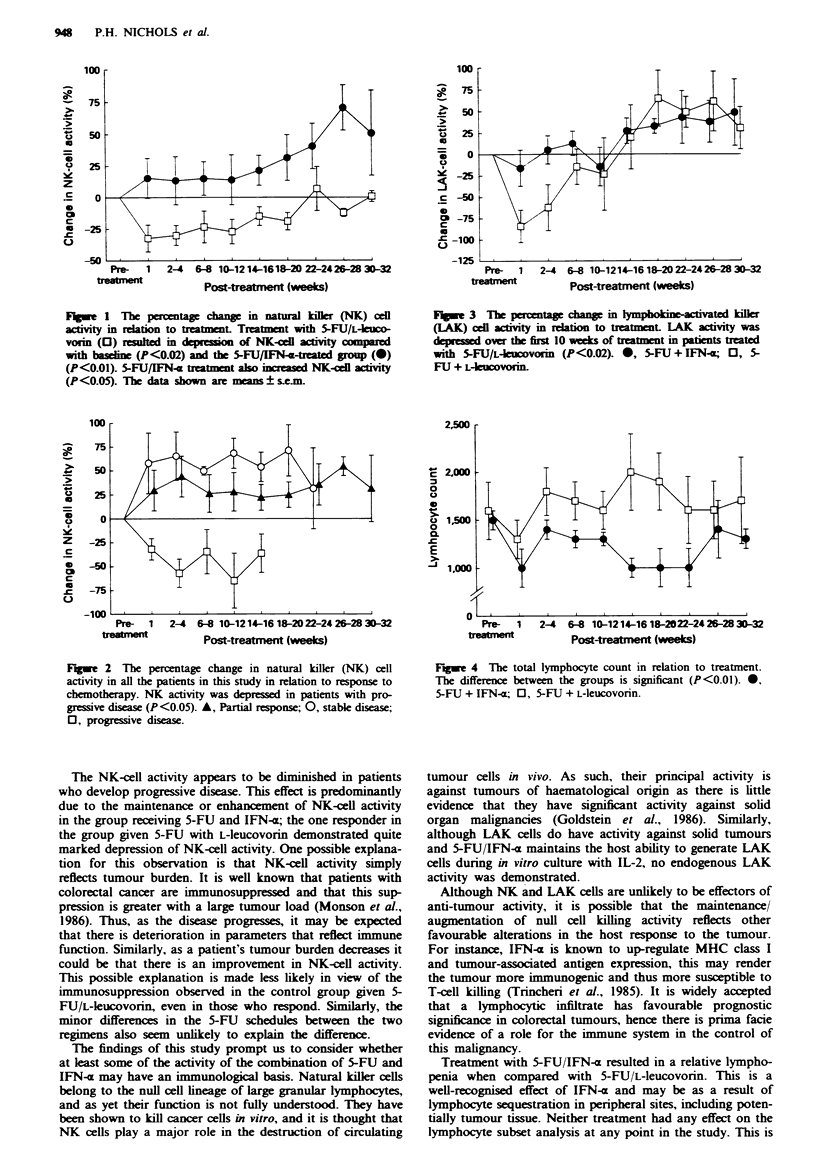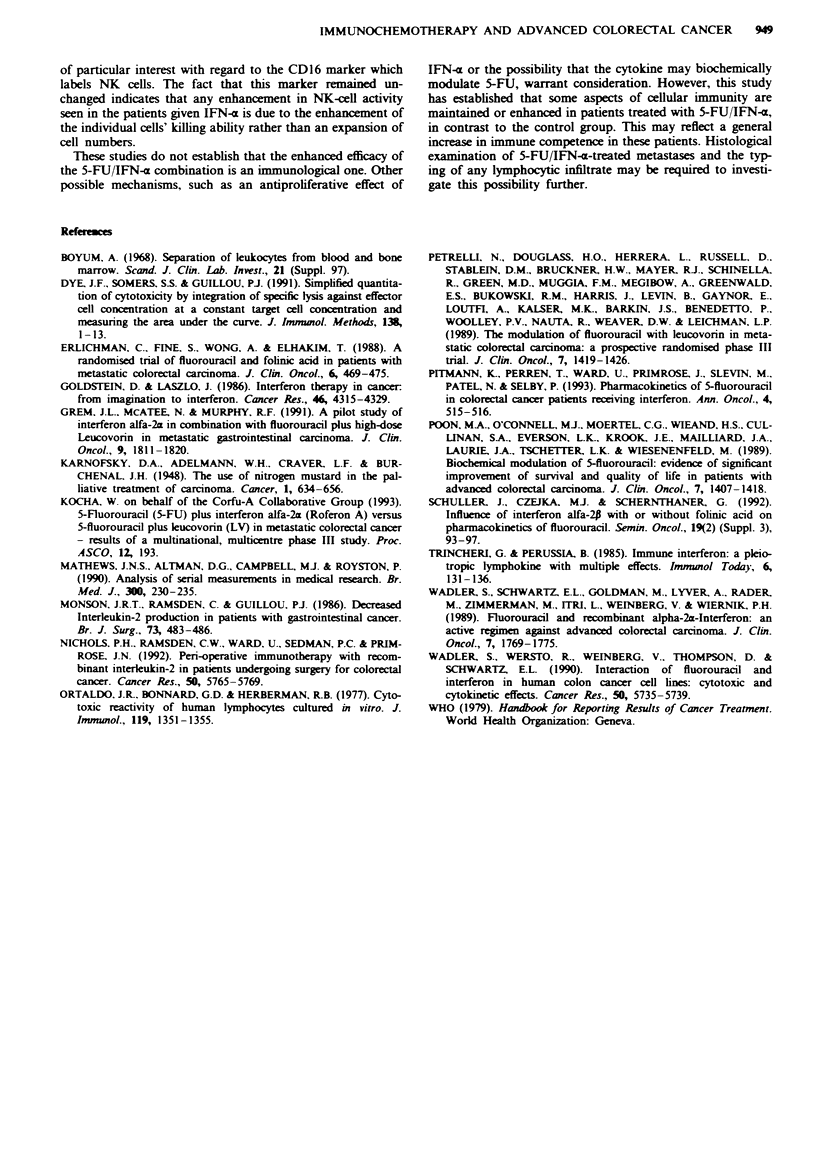# The effect of 5-fluorouracil and alpha interferon and 5-fluorouracil and leucovorin on cellular anti-tumour immune responses in patients with advanced colorectal cancer.

**DOI:** 10.1038/bjc.1994.426

**Published:** 1994-11

**Authors:** P. H. Nichols, U. Ward, C. W. Ramsden, J. N. Primrose

**Affiliations:** Department of Clinical Medicine, St James's University Hospital, Leeds, UK.

## Abstract

Interferon alpha (IFN-alpha) enhances the activity of 5-fluorouracil (5-FU) in the treatment of advanced colorectal cancer although the mechanism is not understood. We have investigated the effect of this combination on cellular immunity and compared this with standard therapy of 5-FU/L-leucovorin, in 24 patients with advanced colorectal cancer. This study has demonstrated an enhancement of the cellular immune response in patients given 5-FU/IFN-alpha with augmentation of natural killer (NK) cell function and abrogation of 5-FU-induced suppression of lymphokine-activated killer (LAK) cell activity.


*Br. J. Cancer* (1994), **70**, 946–949 © Macmillan Press Ltd., 1994

### The effect of 5-fluorouracil and alpha interferon and 5-fluorouracil and leucovorin on cellular anti-tumour immune responses in patients with advanced colorectal cancer

P.H. Nichols, U. Ward, C.W. Ramsden & J.N. Primrose

*Academic Surgical Unit, Department of Clinical Medicine, St James's University Hospital, Beckett Street, Leeds LS9 7TF, UK.*

**Summary** Interferon α (IFN-α) enhances the activity of 5-fluorouracil (5-FU) in the treatment of advanced colorectal cancer although the mechanism is not understood. We have investigated the effect of this combination on cellular immunity and compared this with standard therapy of 5-FU/L-leucovorin, in 24 patients with advanced colorectal cancer. This study has demonstrated an enhancement of the cellular immune response in patients given 5-FU/IFN-α with augmentation of natural killer (NK) cell function and abrogation of 5-FU-induced suppression of lymphokine-activated killer (LAK) cell activity.

The combination of 5-fluorouracil (5-FU) and alpha interferon (IFN-α) is used increasingly in the treatment of advanced colorectal cancer. The initial phase II trial using this combination achieved objective responses in 60% of patients (Wadler *et al.*, 1989) and, although a large phase III study failed to demonstrate such impressive results (Kocha, 1993), they were, nevertheless similar to results with 5-FU and L-leucovorin. 5-FU and L-leucovorin is considered the standard treatment for advanced colorectal cancer, having achieved partial response rates in approximately 40% of patients with modest survival benefit (Petrelli *et al.*, 1989; Poon *et al.*, 1989).

The mechanism by which IFN-α modulates the activity of 5-FU is unknown. *In vitro* cytoxicity studies using gastrointestinal cell lines how that interferon acts synergistically with 5-FU (Wadler *et al.*, 1990). It may be acting as an antiproliferative agent, either by biochemically modulating the effect of 5-FU or by some other mechanism. While other reports suggest that interferon alters the pharmacokinetics of 5-FU (Grem *et al.*, 1991; Schuller *et al.*, 1992) we have demonstrated that the steady-state plasma levels of 5-FU during two 5-day infusions, with and without IFN-α in the same patients, showed no significant differences (Pitman *et al.*, 1993).

Another possibility is that IFN-α may be acting through an immunological mechanism. It is known to augment natural killer (NK) cell activity, and it up-regulates expression of both class I MHC antigen and tumour-associated antigen in tumours (Trincheri *et al.*, 1985), perhaps with the effect of making the tumour more immunogenic. However, little is known about the immunological effects of 5-FU when combined with IFN-α. We have, therefore, studied the effects of this combination on several aspects of cellular immune function in patients with advanced colorectal cancer. The results are compared with those of a control group treated with the conventional therapy of 5-FU and L-leucovorin.

### Materials and methods

### *Patients*

Twenty-four patients were studied, 15 male and nine female. The mean ages were 58 (range 27–76) for the control group and 60 (42–78) for those receiving 5-FU with IFN-α. All patients had histologically proven metastatic colorectal cancer, the sites of metastases being shown in Table I. The time interval between presentation with the primary tumour and recurrent disease averaged 16 (range 0–91 months). Performance status was assessed by means of the Karnofsky scale (Karnofsky *et al.*, 1948), and averaged 80 with a range of 70–90.

Written consent was obtained prior to study entry. The study was approved by Leeds East District Clinical Research (Ethics) Committee.

### *Treatment schedule*

Patients were randomised independently of the authors to receive either 5-FU/IFN-α or 5-FU/L-leucovorin. Fifteen patients were treated with the 5-FU/IFN-α regimen as first reported by Wadler *et al.* (1989). 5-FU was administered as a continuous intravenous infusion for 5 days at a dose of 750 mg $m^{-2}$ $day^{-1}$. This was followed by a weekly bolus dose of 750 mg $m^{-2}$ commencing on day 15. Interferon alpha-2α 9 MU (Roferon-A; Roche Products, Basle, Switzerland) was administered as a subcutaneous injection three times weekly. Nine patients received 5-FU and leucovorin. L-leucovorin, 200 mg $m^{-2}$, was infused over 10 min and followed within 5 min by an intravenous bolus dose of 5-FU at 370 mg $m^{-2}$ for five consecutive days. This cycle was repeated every 4 weeks (Erlichman *et al.*, 1988). There were no differences between the study and control groups in terms of age, sex, burden or distribution of disease or performance status.

### *Tumour assessment*

Serial computerised tomographic (CT) scanning was performed within 2 weeks prior to starting treatment, and at 8

**Table I** The characteristics of the patients in both arms of the study, the sites of metastasis and the response to treatment

| | *5-FU + leucovorin* | *5-FU + IFN-α* |
|---|---|---|
| *n* | 9 | 15 |
| Mean age (range) | 60 (42–79) | 58 (28–76) |
| Male:Female | 5:4 | 10:5 |
| Metastatic site: | | |
| Liver | 3 | 8 |
| Lung | 1 | 1 |
| Liver + lung | 4 | 3 |
| Peritoneal | 1 | 3 |
| Response to treatment: | | |
| Complete response | 0 | 0 |
| Partial response | 1 | 6 |
| Stable disease | 3 | 4 |
| Progressive disease | 5 | 5 |

Correspondence: J.N. Primrose, University Surgical Unit, Tremona Rd, Southampton S09 4XY, UK.
Received 29 November 1993; and in revised form 9 June 1994.

weekly intervals thereafter to assess response. Tumour response was graded in accordance with WHO criteria as described in WHO (1979).

*Immunological studies*

Baseline assessment of lymphocyte number and function was performed before and after the first week of treatment, and subsequently at 4 weekly intervals prior to the next cycle of chemotherapy. For each analysis, a sample of peripheral venous blood was obtained between 08.30 h and 09.30 h to minimise the influence of diurnal variation and the following measurements performed.

*Lymphocyte separation*

Peripheral blood mononuclear cells (PBMCs) were separated from heparinised blood by Lymphoprep (Nycomed Pharma, Oslo, Norway) density-gradient centrifugation after the method of Boyum (1968) and resuspended in complete medium as previously described (Nichols *et al.*, 1992). The cells were counted in a Neubauer counting chamber and diluted to the cell density required for each experiment.

*Cytotoxicity assay*

A standard 4 h chromium-release assay was used to assess cell cytotoxicity (Ortaldo *et al.*, 1997). Freshly isolated PBMCs were used for determination of NK-cell activity with the cell line K562 as target. LAK cells were generated by the co-culture of $15 \times 10^6$ fresh PBMCs with 1,000 units $ml^{-1}$ recombinant interleukin 2 (rIL-2) in 10 ml of complete medium. Cells were incubated for 3 days at 37°C in a humidified atmosphere containing 5% carbon dioxide, prior to use as effectors in the cytotoxicity assay. This was performed in the same way as for the NK assay but using two NK-resistant cell lines as targets, DAUDI (a reference target for LAK cell activity) and COLO 205 (a colonic adenocarcinoma cell line). For details of experimental method see Nichols *et al.* (1992).

To standardise cytolytic activity, results were derived from the area under the curve (AUC) of the log(effector)/response curve exactly as described by Dye *et al.* (1991).

*Cell-surface markers*

Enumeration of leucocyte subpopulations was performed by flow cytometry using a panel of directly conjugated monoclonal antibodies directed against cell-surface antigens. EDTA-stored blood was labelled using a whole-blood technique (Nichols *et al.*, 1992). The following monoclonal antibodies were obtained from Dako (High Wycombe, UK): UCHT1 (CD3, total T cells), MT310 (CD4, helper/inducer T cells), DK25 (CD8, cytotoxic T cells), ACT-1 (CD25, p55 subunit IL-2 receptor), UCHL1 (CD45RO, T-cell activation marker) and mouse IgG1 isotype controls. Anti-Leu-11c (CD16, NK cells) was obtained from Becton Dickinson Immunocytometry Systems. All antibodies were directly conjugated with either fluorescein isothiocyanate (FITC) or phycoerythrin (PE).

Cell preparations were analysed on a Becton Dickinson FACScan analytical flow cytometer. Analysis was performed using 'Lysis II' software (Becton Dickinson) and results determined by four-quadrant analysis having gated for >99% positive cells on isotype controls.

Total leucocyte counts and differential cell counts were performed on a Technicon H1 analyser in the pathology department of St James's University Hospital.

*Statistical analysis*

Statistical analysis was performed in accordance with Matthews *et al.* (1990). In brief, for all the variables of interest individual curves were drawn to establish the pattern of the response. This allowed the identification of a single summary measure for each individual to be used in the analysis. Comparisons between these summary measures were then made using a Student *t*-test. Results are expressed as mean ± s.e.m. with a probability value of <0.05 regarded as statistically significant.

### Results

*Patient outcome*

Response to treatment is shown in Table I. There were no complete responses in either group, and no statistically significant differences in the number of partial responders: 1/9 in the control group and 5/14 in the IFN-α-treated group.

*Treatment-related complications*

Treatment was tolerated well in both treatment groups, with no dose reduction or interruption in therapy necessary. All patients who entered the study were evaluable.

*Lymphocyte function analysis*

Figure 1 demonstrates the percentage change in NK-cell function between the two treatment groups. NK-cell function (arbitrary units) was significantly reduced in the control group following the first week of chemotherapy from 151.7 ± 19.6 to 121.3 ± 16.7. The level of activity failed to return to pretreatment values prior to the next cycle of therapy (122.9 ± 17.9). Using the summary analysis described, the depression in NK-cell activity was significant ($P$<0.02). In contrast, in those patients receiving IFN-α with 5-FU, NK-cell activity was augmented, increasing consistently from a baseline of 117.0 ± 12.9 to 123.7 ± 14.8 after 1 week and to 183.0 ± 16.9 after 26–28 weeks ($P$<0.05). There was a marked difference between the two groups over the first 12 weeks of treatment ($P$<0.01). NK-cell activity was seen to vary in relation to patient outcome, as shown in Figure 2.

The percentage change in LAK-cell generation against the DAUDI target is shown in Figure 3. As with NK-cell activity, LAK-cell function was reduced following treatment with 5-FU an L-leucovorin, from a pretreatment value of 265.4 ± 18.8 to 170.1 ± 30.2. Again activity remained reduced prior to the next treatment cycle, 201.4 ± 23.4, although in the longer term LAK-cell activity did return to pretreatment levels. The depression in the LAK-cell activity in the first 10 weeks of treatment was statistically significant ($P$<0.02). In contrast, treatment with 5-FU and IFN-α resulted in no such fall in LAK-cell generative capacity.

*Lymphocyte phenotypic analysis*

We failed to show any changes in the phenotypic pattern of peripheral blood lymphocytes throughout our monitoring period for both groups of patients (results not shown). Numbers of circulating lymphocytes were, however, reduced in those patients receiving IFN-α but not L-leucovorin ($P$<0.01) (Figure 4).

### Discussion

This study demonstrates that 5-FU combined with L-leucovorin, the most widely used modulated 5-FU regimen, has a predominantly immunosuppressive effect. Both NK- and LAK-cell activity were diminished after the first cycle of treatment and did not return to the baseline level before the next cycle. The NK-cell activity remained suppressed compared with that in the 5-FU/IFN-α-treated group for a prolonged period. By contrast, the interferon-treated group showed no suppression of NK- or LAK-cell activity, and NK-cell activity was augmented.

**Figure 1** The percentage change in natural killer (NK) cell activity in relation to treatment. Treatment with 5-FU/L-leucovorin (□) resulted in depression of NK-cell activity compared with baseline ($P<0.02$) and the 5-FU/IFN-α-treated group (●) ($P<0.01$). 5-FU/IFN-α treatment also increased NK-cell activity ($P<0.05$). The data shown are means ± s.e.m.

**Figure 3** The percentage change in lymphokine-activated killer (LAK) cell activity in relation to treatment. LAK activity was depressed over the first 10 weeks of treatment in patients treated with 5-FU/L-leucovorin ($P<0.02$). ●, 5-FU + IFN-α; □, 5-FU + L-leucovorin.

**Figure 2** The percentage change in natural killer (NK) cell activity in all the patients in this study in relation to response to chemotherapy. NK activity was depressed in patients with progressive disease ($P<0.05$). ▲, Partial response; O, stable disease; □, progressive disease.

**Figure 4** The total lymphocyte count in relation to treatment. The difference between the groups is significant ($P<0.01$). ●, 5-FU + IFN-α; □, 5-FU + L-leucovorin.

The NK-cell activity appears to be diminished in patients who develop progressive disease. This effect is predominantly due to the maintenance or enhancement of NK-cell activity in the group receiving 5-FU and IFN-α; the one responder in the group given 5-FU with L-leucovorin demonstrated quite marked depression of NK-cell activity. One possible explanation for this observation is that NK-cell activity simply reflects tumour burden. It is well known that patients with colorectal cancer are immunosuppressed and that this suppression is greater with a large tumour load (Monson et al., 1986). Thus, as the disease progresses, it may be expected that there is deterioration in parameters that reflect immune function. Similarly, as a patient's tumour burden decreases it could be that there is an improvement in NK-cell activity. This possible explanation is made less likely in view of the immunosuppression observed in the control group given 5-FU/L-leucovorin, even in those who respond. Similarly, the minor differences in the 5-FU schedules between the two regimens also seem unlikely to explain the difference.

The findings of this study prompt us to consider whether at least some of the activity of the combination of 5-FU and IFN-α may have an immunological basis. Natural killer cells belong to the null cell lineage of large granular lymphocytes, and as yet their function is not fully understood. They have been shown to kill cancer cells *in vitro*, and it is thought that NK cells play a major role in the destruction of circulating tumour cells *in vivo*. As such, their principal activity is against tumours of haematological origin as there is little evidence that they have significant activity against solid organ malignancies (Goldstein et al., 1986). Similarly, although LAK cells do have activity against solid tumours and 5-FU/IFN-α maintains the host ability to generate LAK cells during *in vitro* culture with IL-2, no endogenous LAK activity was demonstrated.

Although NK and LAK cells are unlikely to be effectors of anti-tumour activity, it is possible that the maintenance/augmentation of null cell killing activity reflects other favourable alterations in the host response to the tumour. For instance, IFN-α is known to up-regulate MHC class I and tumour-associated antigen expression, this may render the tumour more immunogenic and thus more susceptible to T-cell killing (Trincheri et al., 1985). It is widely accepted that a lymphocytic infiltrate has favourable prognostic significance in colorectal tumours, hence there is prima facie evidence of a role for the immune system in the control of this malignancy.

Treatment with 5-FU/IFN-α resulted in a relative lymphopenia when compared with 5-FU/L-leucovorin. This is a well-recognised effect of IFN-α and may be as a result of lymphocyte sequestration in peripheral sites, including potentially tumour tissue. Neither treatment had any effect on the lymphocyte subset analysis at any point in the study. This is of particular interest with regard to the CD16 marker which labels NK cells. The fact that this marker remained unchanged indicates that any enhancement in NK-cell activity seen in the patients given IFN-α is due to the enhancement of the individual cells' killing ability rather than an expansion of cell numbers.

These studies do not establish that the enhanced efficacy of the 5-FU/IFN-α combination is an immunological one. Other possible mechanisms, such as an antiproliferative effect of IFN-α or the possibility that the cytokine may biochemically modulate 5-FU, warrant consideration. However, this study has established that some aspects of cellular immunity are maintained or enhanced in patients treated with 5-FU/IFN-α, in contrast to the control group. This may reflect a general increase in immune competence in these patients. Histological examination of 5-FU/IFN-α-treated metastases and the typing of any lymphocytic infiltrate may be required to investigate this possibility further.

### References

BOYUM, A. (1968). Separation of leukocytes from blood and bone marrow. *Scand. J. Clin. Lab. Invest.*, **21** (Suppl. 97).

DYE, J.F., SOMERS, S.S. & GUILLOU, P.J. (1991). Simplified quantitation of cytotoxicity by integration of specific lysis against effector cell concentration at a constant target cell concentration and measuring the area under the curve. *J. Immunol. Methods*, **138**, 1–13.

ERLICHMAN, C., FINE, S., WONG, A. & ELHAKIM, T. (1988). A randomised trial of fluorouracil and folinic acid in patients with metastatic colorectal carcinoma. *J. Clin. Oncol.*, **6**, 469–475.

GOLDSTEIN, D. & LASZLO, J. (1986). Interferon therapy in cancer: from imagination to interferon. *Cancer Res.*, **46**, 4315–4329.

GREM, J.L., MCATEE, N. & MURPHY, R.F. (1991). A pilot study of interferon alfa-2α in combination with fluorouracil plus high-dose Leucovorin in metastatic gastrointestinal carcinoma. *J. Clin. Oncol.*, **9**, 1811–1820.

KARNOFSKY, D.A., ADELMANN, W.H., CRAVER, L.F. & BURCHENAL, J.H. (1948). The use of nitrogen mustard in the palliative treatment of carcinoma. *Cancer*, **1**, 634–656.

KOCHA, W. on behalf of the Corfu-A Collaborative Group (1993). 5-Fluorouracil (5-FU) plus interferon alfa-2α (Roferon A) versus 5-fluorouracil plus leucovorin (LV) in metastatic colorectal cancer – results of a multinational, multicentre phase III study. *Proc. ASCO*, **12**, 193.

MATHEWS, J.N.S., ALTMAN, D.G., CAMPBELL, M.J. & ROYSTON, P. (1990). Analysis of serial measurements in medical research. *Br. Med. J.*, **300**, 230–235.

MONSON, J.R.T., RAMSDEN, C. & GUILLOU, P.J. (1986). Decreased Interleukin-2 production in patients with gastrointestinal cancer. *Br. J. Surg.*, **73**, 483–486.

NICHOLS, P.H., RAMSDEN, C.W., WARD, U., SEDMAN, P.C. & PRIMROSE, J.N. (1992). Peri-operative immunotherapy with recombinant interleukin-2 in patients undergoing surgery for colorectal cancer. *Cancer Res.*, **50**, 5765–5769.

ORTALDO, J.R., BONNARD, G.D. & HERBERMAN, R.B. (1977). Cytotoxic reactivity of human lymphocytes cultured *in vitro*. *J. Immunol.*, **119**, 1351–1355.

PETRELLI, N., DOUGLASS, H.O., HERRERA, L., RUSSELL, D., STABLEIN, D.M., BRUCKNER, H.W., MAYER, R.J., SCHINELLA, R., GREEN, M.D., MUGGIA, F.M., MEGIBOW, A., GREENWALD, E.S., BUKOWSKI, R.M., HARRIS, J., LEVIN, B., GAYNOR, E., LOUTFI, A., KALSER, M.K., BARKIN, J.S., BENEDETTO, P., WOOLLEY, P.V., NAUTA, R., WEAVER, D.W. & LEICHMAN, L.P. (1989). The modulation of fluorouracil with leucovorin in metastatic colorectal carcinoma: a prospective randomised phase III trial. *J. Clin. Oncol.*, **7**, 1419–1426.

PITMANN, K., PERREN, T., WARD, U., PRIMROSE, J., SLEVIN, M., PATEL, N. & SELBY, P. (1993). Pharmacokinetics of 5-fluorouracil in colorectal cancer patients receiving interferon. *Ann. Oncol.*, **4**, 515–516.

POON, M.A., O'CONNELL, M.J., MOERTEL, C.G., WIEAND, H.S., CULLINAN, S.A., EVERSON, L.K., KROOK, J.E., MAILLIARD, J.A., LAURIE, J.A., TSCHETTER, L.K. & WIESENENFELD, M. (1989). Biochemical modulation of 5-fluorouracil: evidence of significant improvement of survival and quality of life in patients with advanced colorectal carcinoma. *J. Clin. Oncol.*, **7**, 1407–1418.

SCHULLER, J., CZEJKA, M.J. & SCHERNTHANER, G. (1992). Influence of interferon alfa-2β with or without folinic acid on pharmacokinetics of fluorouracil. *Semin. Oncol.*, **19**(2) (Suppl. 3), 93–97.

TRINCHERI, G. & PERUSSIA, B. (1985). Immune interferon: a pleiotropic lymphokine with multiple effects. *Immunol Today*, **6**, 131–136.

WADLER, S., SCHWARTZ, E.L., GOLDMAN, M., LYVER, A., RADER, M., ZIMMERMAN, M., ITRI, L., WEINBERG, V. & WIERNIK, P.H. (1989). Fluorouracil and recombinant alpha-2α-Interferon: an active regimen against advanced colorectal carcinoma. *J. Clin. Oncol.*, **7**, 1769–1775.

WADLER, S., WERSTO, R., WEINBERG, V., THOMPSON, D. & SCHWARTZ, E.L. (1990). Interaction of fluorouracil and interferon in human colon cancer cell lines: cytotoxic and cytokinetic effects. *Cancer Res.*, **50**, 5735–5739.

WHO (1979). *Handbook for Reporting Results of Cancer Treatment*. World Health Organization: Geneva.